# Long-Term Safety and Efficacy of Blonanserin Oral Tablet in Adolescents with Schizophrenia: A 52-Week, Multicenter, Open-Label Extension Study

**DOI:** 10.1089/cap.2021.0058

**Published:** 2022-02-14

**Authors:** Takuya Saito, Yohei Hyodo, Reiko Sakaguchi, Hiroshi Nakamura, Jun Ishigooka

**Affiliations:** ^1^ Department of Child and Adolescent Psychiatry, Hokkaido University Hospital, Sapporo, Japan.; ^2^ Sumitomo Dainippon Pharma Co., Ltd., Chuo-Ku, Tokyo, Japan.; ^3^ Institute of CNS Pharmacology, Shibuya-ku, Tokyo, Japan.

**Keywords:** adolescent, antipsychotics, blonanserin, schizophrenia

## Abstract

***Objectives:*** To evaluate the long-term efficacy and safety/tolerability of oral blonanserin in adolescents with schizophrenia (Study registration number: JapicCTI-111725).

***Methods:*** This 52-week, multicenter, open-label extension study enrolled adolescent patients with schizophrenia who opted to enter in this study after the completion of the preceding placebo-controlled study. Blonanserin tablet was orally administered twice daily, after morning and evening meals, for 52 weeks using dose-titration method within a range between 4 and 24 mg/day. The primary end point was the change from baseline to the end of the study in the Positive and Negative Syndrome Scale (PANSS) total score. Safety/tolerability was assessed by the incidence and severity of adverse events.

***Results:*** Of 117 patients who completed the preceding placebo-controlled study, 109 entered this extension study and 43 (39.4%) of them discontinued the study treatment. The safety analysis set comprised 106 patients who received the study drug at least once, including 36 and 70 patients treated with placebo (DB-placebo group) and blonanserin tablet (DB-blonanserin group), respectively, in the placebo-controlled study. At the last assessment, the mean change in PANSS total score overall [mean (standard deviation)] was −24.9 (20.76) from the baseline of the placebo-controlled study, which was similar in the DB-placebo and DB-blonanserin groups. The overall incidence of adverse events was 90.6%, and most of them were mild or moderate in severity, with similar incidence of extrapyramidal symptoms (38.7%) to that in adults receiving long-term blonanserin oral tablet treatment and minimal change in weight and metabolic parameters.

***Conclusions:*** This long-term extension study showed that 52 weeks of oral blonanserin treatment improved or stabilized psychiatric symptoms in patients with adolescent schizophrenia. There were no major issues with the safety or tolerability of blonanserin administration in this study. Considering relatively less adverse effects on weight increase and metabolic parameters, blonanserin is expected to be a safe/tolerable treatment option for adolescent schizophrenia that can be used seamlessly from adolescence to adulthood.

## Introduction

In recent years, atypical antipsychotics have been approved for indications of adolescent schizophrenia. Guidelines for treatment of schizophrenia have recommended the use of atypical antipsychotics, with consideration of the balance between effectiveness and safety/tolerability, as drug therapy for schizophrenia spectrum disorders in children (McClellan et al. 2013; Abidi et al. 2017).

Meta-analyses of randomized controlled studies in patients with adolescent schizophrenia showed the efficacy of second-generation antipsychotics and demonstrated the differences among the effects of these agents on the improvement of psychiatric symptoms, weight increased, and withdrawal rate (Pagsberg et al. 2017; Krause et al. 2018). In selecting a drug, therefore, it is important to balance its effectiveness and profile of side effects (Harvey et al. 2016). Considering that the risk of relapse increases when patients with stable first-episode psychosis discontinue the use of antipsychotics (Kishi et al. 2019), such patients need drug therapy that can be continued seamlessly from adolescence to adulthood. However, to ensure the continuation of long-term administration of antipsychotics from adolescence into adulthood, it is essential to confirm not only sustained effectiveness but also tolerability because adolescent patients are vulnerable to adverse events (McClellan et al. 2013).

Blonanserin, an atypical antipsychotic, is a selective antagonist of dopamine D_2_, D_3_, and serotonin 5-HT_2A_ receptors. Its efficacy and safety have been confirmed in adult patients with schizophrenia (Murasaki 2007a; Miura 2008; Garcia et al. 2009; Harvey et al. 2019, 2020), and long-term administration has not shown any clinical issues regarding maintenance of effect or tolerability (Murasaki 2007b; Kinoshita 2008; Deeks and Keating 2010). In a 6-week, multicenter, placebo-controlled, randomized, double-blind, parallel-group study of patients with adolescent schizophrenia (aged 12–18 years who were diagnosed with schizophrenia according to the Diagnostic and Statistical Manual of Mental Disorders, 4th Edition, Text Revision [DSM-IV-TR] (American Psychiatric Association, 2000) and confirmed with the Mini international neuropsychiatric interview for children and adolescents), we confirmed the efficacy of blonanserin compared with placebo; the safety was not considerably different from that in adult patients, with no noteworthy clinical risks observed (reference: submitted for publication; adolescent placebo-controlled study).

We reported the results of a 52-week, long-term, open-label extension study in continuation of the preceding placebo-controlled study; we evaluated the sustained effectiveness and safety/tolerability of extended blonanserin use in patients with adolescent schizophrenia.

## Methods

### Patients

The patients with adolescent schizophrenia opted to participate in this study after the completion of the placebo-controlled study. The main inclusion criteria of the preceding placebo-controlled study were: 12–18 years of age; diagnosis with schizophrenia according to the DSM-IV-TR confirmed with the Mini international neuropsychiatric interview for children and adolescents; a total score of 60–120 in the Positive and Negative Syndrome Scale (PANSS); and an assessment score of at least 3 (mildly ill) in the Clinical Global Impressions-Severity of Illness Scale (CGI-S). The main exclusion criteria of this study were: contraindications for oral blonanserin; concurrent or previous malignant syndrome, tardive dyskinesia, paralytic ileus, rhabdomyolysis, agranulocytosis, pulmonary embolism, or deep vein thrombosis; Parkinson's disease; strong suicidal ideation; diabetes mellitus; concurrent diseases such as serious cardiovascular disease, liver disease, kidney disease, organic brain disease, hematologic disease, endocrine disease, or spastic disease; a history of substance abuse or dependence, alcohol abuse or dependence; and actual or possible pregnancy.

This study was approved in advance by the institutional review boards of all the participating medical institutions and conducted in accordance with the ethical principles of the Declaration of Helsinki and in line with regulatory requirements, including Japan's ministerial ordinance on Good Clinical Practice. After explanation of all aspects of the study, written assent was obtained from all the patients, and consent was obtained from their parents/legal guardians.

### Study design

**FIG. 1.**
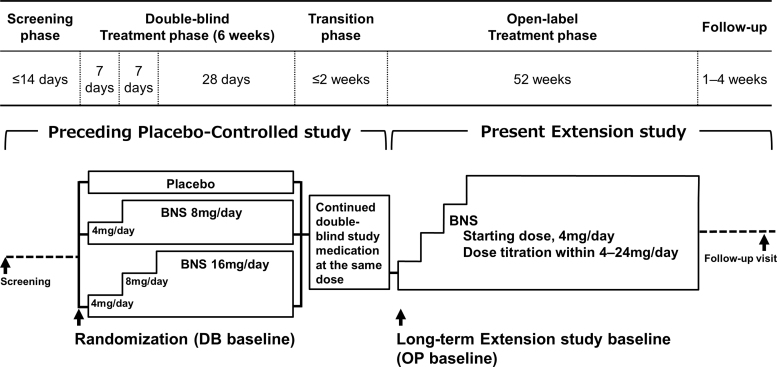
Study schematic. Patients who opted to enter in this study after the completion of the preceding placebo-controlled study were enrolled. Blonanserin tablet was orally administered twice daily, after morning and evening meals, for 52 weeks using dose-titration method within a range between 4 and 24 mg/day. BNS, blonanserin.

This multicenter, open-label extension study was conducted between April 2012 and March 2020 at 72 medical institutions in Japan (clinical study No.: JapicCTI-111725). A dose-titration method was used in patients who opted to participate in this study after the completion of the preceding double-blind, placebo-controlled study on patients between the ages of 12 and 18 years with adolescent schizophrenia (clinical study No.: JapicCTI-111724; a 6-week, randomized placebo-controlled study). Blonanserin tablet was orally administered twice daily, after morning and evening meals, for 52 weeks. Medication adherence was calculated as total dose actually taken divided by total dose that should have been taken based on pill count. The dose was 4 mg/day during the first week, after which it was adjusted as necessary within a range between 4 and 24 mg/day. A single dose did not exceed 12 mg; when a response to single doses lower than 12 mg was insufficient, the dose was increased by 2–4 mg per dose if there were no tolerability issues (Fig. 1).

### Concomitant medications and treatments

Concomitant use of antipsychotics other than the study drug was prohibited for the duration of 52 weeks from the start of drug administration or for patients who withdrew from the study until the completion of the last assessment. However, in situations such as sudden psychomotor agitation that required emergency intervention, the use of a single antipsychotic in nontablet formulation (other than a depot preparation) was allowed as needed but was not to be used within 48 hours of efficacy assessments (limited to a total of 10 days of use during the study period).

Patients who had used antiparkinsonian agents, psychotropics (mainly anxiolytic), or hypnotics at the end of the placebo-controlled study were allowed to continue using the drugs at the same dosage and administration. Starting these drugs or increasing the dose was allowed if a new adverse event occurred or worsened. The administration of psychotropics and hypnotics was prohibited within 12 hours before each efficacy evaluation.

Drug therapies for concurrent conditions (e.g., hypertension, dyslipidemia) were continued without changing the dosage and administration unless the conditions worsened or improved. The use of antimanic or antiepileptic agents, MAO inhibitors, CYP3A4 inhibitors (except topical skin agents), epinephrine (except when used as emergency intervention for anaphylaxis), and other study drugs or postmarketing clinical study drugs, as well as electroconvulsive therapy, was prohibited.

### Efficacy assessments

Efficacy was assessed at weeks 1, 2, 4, 8, 12, 16, 20, 24, 28, 36, 44, and 52 using PANSS, CGI-S, and Clinical Global Impressions-Improvement Scale (CGI-I). The primary efficacy end point was the change in PANSS (Kay et al. 1987) total score at the last assessment from the start of the preceding placebo-controlled study (DB-baseline) and from the start of the present extension study (OP-baseline). Secondary end points were the change in PANSS total score at each assessment point, change in PANSS subscale scores at the last assessment and at each assessment point (Perkins et al. 2000), change in PANSS 5-factor model scores (Lindenmayer et al. 1994), change in PANSS symptom scores, PANSS remission rate (Andreasen et al. 2005), percentage of PANSS responders (Leucht et al. 2017), change in CGI-S score (Guy 1976), rate of improvement in CGI-I (the percentage of patients with CGI-I of 1 [very much improved] or 2 [much improved]), and number of days from the first to the last administration of the study drug (treatment continuation rate). PANSS assessments were performed by the assessors who were trained and certified.

### Pharmacokinetics assessments

Plasma concentration of blonanserin and concentration of the metabolite M-1 (N-demethylated form) were measured for pharmacokinetic assessment. Samples for pharmacokinetic assessment were collected at weeks 28 and 52.

### Safety assessments

Safety was assessed by examining incidence and severity of adverse events. Adverse events were recorded and classified in accordance with the version 21.1 of the Medical Dictionary for Regulatory Activities of the International Council for Harmonisation of Technical Requirements for Pharmaceuticals for Human Use.

Extrapyramidal symptoms were evaluated by examining change in total score on the Drug-Induced Extrapyramidal Symptoms Scale (DIEPSS) (Inada et al. 2003) and percentage of patients using antiparkinsonian agents at the last assessment and at each assessment point. DIEPSS is a physician-rating scale to assess the severity of extrapyramidal symptoms induced by antipsychotics on a 5-rank scale of 0 (normal) to 4 (severe) for each of eight symptom categories (gait, bradykinesia, sialorrhea, muscle rigidity, tremor, akathisia, dystonia, and dyskinesia) and one global assessment (overall severity).

Suicide risk was evaluated by examining the change in Clinical Global Impressions of Suicide Severity (CGI-SS) score and deterioration rate at the last assessment and at each assessment (Meltzer et al. 2003). CGI-SS is an overall clinician-rating scale of the clinical risk of suicidality and change in suicidality. The CGI-SS item 1 has five levels of severity of suicidality in the past 7 days: 1, not at all suicidal; 2, mildly suicidal; 3, moderately suicidal; 4, severely suicidal; 5, attempted suicide. The CGI-SS item 2 has seven levels of change from baseline in suicidality: 1, very much improved; 2, much improved; 3, minimally improved; 4, no change; 5, minimally worsened; 6, much worse; 7, very much worse. The deterioration rate is the percentage of patients whose most recent improvement status was 6 (much worse) or 7 (very much worse) compared with their condition at DB-baseline. Changes in laboratory test values, vital signs, body weight, and body mass index (BMI), as well as electrocardiographic findings and changes in electrocardiographic parameters, were evaluated at the last assessment and at each assessment.

### Statistical analysis

The SAS version 9.4 software was used to perform statistical analysis. The target number of patients was set at up to 150 because the patients were to opt to continue in this study after completion of the placebo-controlled study. All analyses of efficacy and safety were performed in the safety analysis set. The safety analysis set comprised patients who received the study drug at least once after entering the present extension study. The pharmacokinetic analysis set comprised those patients who received blonanserin at least once after entering the present extension study and had analyzable measurements from at least one time point.

Summary statistics of the mean change from baseline was calculated for PANSS and CGI-S scores, whereas subject numbers and percentages were calculated for PANSS score remission rate, percentage of PANSS responders, and rate of improvement in CGI-I. In analysis of the last assessments, last observation carried forward (LOCF) method was used to impute missing values. The LOCF end point was defined as the last nonmissing measurement that satisfied the definition of postbaseline.

For body weight and height, z-scores ([weight or height of subject−mean weight or height at the age and sex]/standard deviation [SD] of weight or height at the age and sex) and percentiles were calculated for each subject from the data on Japanese children stratified by sex and age. Results of Survey of School Health Statistics by Japan's Ministry of Education, Culture, Sports, Science and Technology (2017) (Portal Site of Official Statistics in Japan 2019) and summary statistics of change from baseline were calculated.

## Results

### Patient disposition and baseline characteristics

**FIG. 2.**
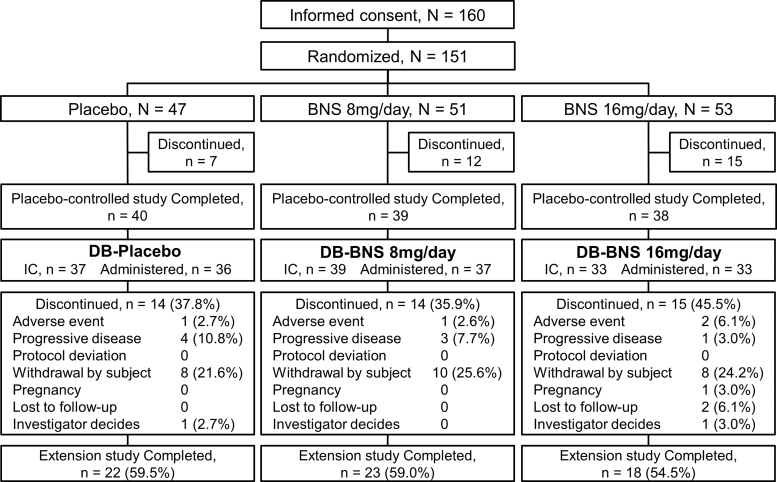
Patient disposition. Of 117 patients who completed the preceding placebo-controlled study, 109 entered this study and 43 (39.4%) of them discontinued the study treatment. The safety analysis set comprised 106 patients who received the study drug at least once, including 36 and 70 patients treated with placebo (DB-placebo group) and blonanserin tablet (DB-blonanserin group), respectively, in the placebo-controlled study. BNS, blonanserin.

Informed consent was obtained from 109 of 117 patients who completed the placebo-controlled study, and 106 patients received the study drug. The percentage of patients from the placebo-controlled study who received the study drug in the long-term extension study was 70.2%. Of 109 subjects who provided informed consent, 63 (57.8%) completed the present extension study, and 43 (39.4%) withdrew from the study. The most common reasons for withdrawal were withdrawal by subject (26 patients), progressive disease (8 patients), and adverse event (4 patients) (Fig. 2).

The overall safety analysis set comprised 106 patients, of which 36 had been allocated to receive the placebo (DB-placebo group), and 70 had been allocated to receive blonanserin tablet (DB-blonanserin group: 37 patients at 8 mg/day; 33 patients at 16 mg/day) in the placebo-controlled study (Fig. 2). The pharmacokinetic analysis set comprised 103 patients. The baseline characteristics of patients in the safety analysis set are shown in Table 1.

**Table 1.** Demographic and Clinical Characteristics in Patients at Baseline (Safety Analysis Set)

	DB-Placebo (*N* = 36)	DB-BNS (*N* = 70)	Overall (*N* = 106)
Sex, male, *n* (%)	14 (38.9)	31 (44.3)	45 (42.5)
Age (years), mean (SD)	15.7 (1.75)	15.5 (1.58)	15.6 (1.63)
Age (years), ≥15, *n* (%)	27 (75.0)	49 (70.0)	76 (71.7)
Height (cm), mean (SD)	160.96 (6.803)	161.93 (7.090)	161.60 (6.976)
Weight (kg), mean (SD)	51.88 (8.025)	58.32 (11.311)	56.13 (10.720)
Weight (kg), ≥50 kg, *n* (%)	21 (58.3)	52 (74.3)	73 (68.9)
BMI (kg/m^2^), mean (SD)	19.99 (2.462)	22.19 (3.776)	21.44 (3.534)
DSM-IV subtype, *n* (%)			
Disorganized	7 (19.4)	6 (8.6)	13 (12.3)
Catatonic	2 (5.6)	11 (15.7)	13 (12.3)
Paranoid	14 (38.9)	29 (41.4)	43 (40.6)
Residual	0	2 (2.9)	2 (1.9)
Undifferentiated	13 (36.1)	22 (31.4)	35 (33.0)
No. of episodes, *n* (%)			
1	29 (80.6)	54 (77.1)	83 (78.3)
≥2	5 (13.9)	15 (21.4)	20 (18.9)
Unknown	2 (5.6)	1 (1.4)	3 (2.8)
Recruitment status,^a^ *n* (%)			
Inpatient	20 (55.6)	41 (58.6)	61 (57.5)
Outpatient	16 (44.4)	29 (41.4)	45 (42.5)
Age at initial diagnosis (years), mean (SD)	13.3 (2.27)	13.0 (2.02)	13.1 (2.10)
Duration of illness (years), mean (SD)	2.24 (1.615)	2.31 (1.669)	2.28 (1.644)
Duration of current episodes (days), mean (SD)	666.9 (647.95)	629.3 (586.21)	642.1 (605.05)
DB baseline PANSS total score, mean (SD)	88.9 (9.24)	87.0 (13.70)	87.7 (12.35)
OP baseline PANSS total score, mean (SD)	73.1 (15.48)	66.4 (15.92)	68.7 (16.01)
PANSS composite subscale at DB baseline, *n* (%)			
Positive subscale score > Negative subscale score	13 (36.1)	30 (42.9)	43 (40.6)
Positive subscale score = Negative subscale score	3 (8.3)	3 (4.3)	6 (5.7)
Positive subscale score < Negative subscale score	20 (55.6)	37 (52.9)	57 (53.8)
PANSS composite subscale at OP baseline, *n* (%)			
Positive subscale score > Negative subscale score	9 (25.0)	23 (32.9)	32 (30.2)
Positive subscale score = Negative subscale score	4 (11.1)	9 (12.9)	13 (12.3)
Positive subscale score < Negative subscale score	23 (63.9)	38 (54.3)	61 (57.5)
DB Baseline CGI-S score, mean (SD)	3.97 (0.696)	4.00 (0.659)	3.99 (0.669)
OP Baseline CGI-S score, mean (SD)	3.28 (0.944)	2.99 (0.737)	3.09 (0.822)
DB Baseline CGI-SS score, mean (SD)	1.11 (0.319)	1.07 (0.259)	1.08 (0.280)
DB Baseline DIEPSS total score, mean (SD)	0.19 (0.822)	0.26 (1.031)	0.24 (0.962)

Treatment group at placebo-controlled study: DB-Placebo = Placebo; DB-BNS = Blonanserin 8 or 16 mg; Overall = Placebo, Blonanserin 8 or 16 mg. DB baseline: Baseline of placebo-controlled study, OP baseline: day 1 of extension study.

^a^ At the time of informed consent in the placebo-controlled study.

BMI, body mass index; CGI-S, Clinical Global Impressions-Severity of Illness Scale; CGI-SS, Clinical Global Impressions of Suicide Severity; DIEPSS, Drug-Induced Extrapyramidal Symptoms Scale; DSM-IV, Diagnostic and Statistical Manual of Mental Disorders, 4th edition; PANSS, Positive and Negative Syndrome Scale; SD, standard deviation.

Overall, the mean adherence rate exceeded 95%, with an adherence rate of at least 80% in 100 patients (94.3%). The treatment continuation rate was 75.5% at week 24 (168 days) and 60.4% at week 52 (364 days). The continuation rates did not greatly differ between the three groups (placebo group, 8 mg/day group, 16 mg/day group) allocated in the placebo-controlled study. The mean modal dose was 9.6 mg/day, with 65 patients (61.3%) in the 8–16 mg/day range, 33 patients (31.1%) receiving <8 mg/day, and 8 patients (7.5%) receiving >16 mg/day. The mean maximum dose was 11.2 mg/day, with 68 patients (64.2%) in the 8–16 mg/day range, 23 patients (21.7%) receiving <8 mg/day, and 15 patients (14.2%) receiving >16 mg/day. The percentage of patients using concomitant medications was as follows: antipsychotics in 8.5%, antiparkinsonian agents in 45.3%, psychotropics in 67.9%, and hypnotics in 75.5% (Table 2).

**Table 2.** Modal and Maximum Daily Dose of Blonanserin and Concomitant Medication Use

	DB-Placebo (*N* = 36)	DB-BNS (*N* = 70)	Overall (*N* = 106)
BNS tablet			
Modal daily dose, mg/day			
Mean (SD)	9.6 (5.30)	9.6 (5.07)	9.6 (5.12)
Median (minimum to maximum)	8.0 (4–24)	8.0 (4–24)	8.0 (4–24)
Modal daily dose, *n* (%)			
<8 mg/day	12 (33.3)	21 (30.0)	33 (31.1)
≥8 to ≤16 mg/day	21 (58.3)	44 (62.9)	65 (61.3)
>16 mg/day	3 (8.3)	5 (7.1)	8 (7.5)
Maximum daily dose, mg/day			
Mean (SD)	11.8 (5.84)	10.9 (5.52)	11.2 (5.62)
Median (minimum to maximum)	12.0 (4–24)	8.0 (4–24)	11.0 (4–24)
Maximum daily dose, *n* (%)			
<8 mg/day	7 (19.4)	16 (22.9)	23 (21.7)
≥8 to ≤16 mg/day	23 (63.9)	45 (64.3)	68 (64.2)
>16 mg/day	6 (16.7)	9 (12.9)	15 (14.2)
Concomitant medications,^a^ *n* (%)			
Antipsychotics^b^	4 (11.1)	5 (7.1)	9 (8.5)
Antiparkinson agents	14 (38.9)	34 (48.6)	48 (45.3)
Psychotropics (antidepressant, anxiolytic, etc.)	26 (72.2)	46 (65.7)	72 (67.9)
Hypnotics	28 (77.8)	52 (74.3)	80 (75.5)

^a^ Concomitant medications were classified according to WHO Drug Dictionary version September 1, 2017. If a patient used multiple medications in the same medication category, the patient was counted only once for the category.

^b^ Concomitant antipsychotics was prohibited from the start of study drug administration until the completion of the last assessment during the treatment period. However, in situations such as sudden psychomotor agitation requiring emergency intervention, use of a single antipsychotic in nontablet formulation (other than a depot preparation) was allowed as needed but limited to a total 10 days of use during the study period.

BNS, blonanserin; SD, standard deviation.

### Efficacy

**FIG. 3.**
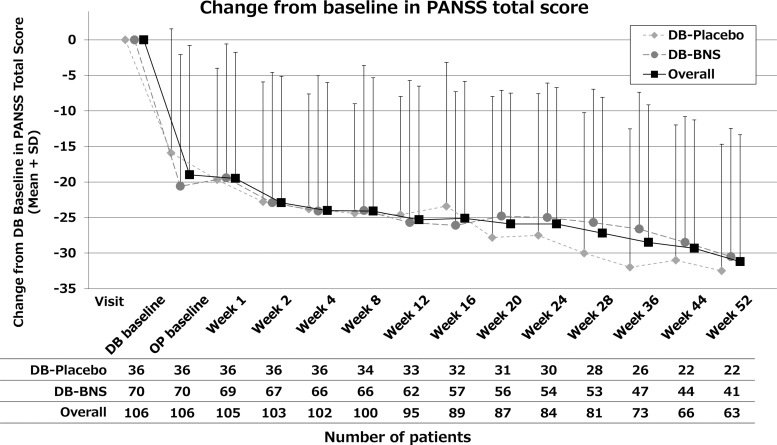
Change from double-blind baseline in PANSS total score. DB-Placebo: patients allocated to placebo treatment in the precedent placebo-controlled study. DB-BNS: patients allocated to blonanserin treatment in the precedent placebo-controlled study. BNS, blonanserin; PANSS, Positive and Negative Syndrome Scale; SD, standard deviation.

At the last assessment (LOCF), the mean change in PANSS total score overall [mean (SD)] was −24.9 (20.76) from DB-baseline and −6.0 (15.77) from OP-baseline. The score was maintained from OP-baseline without worsening. While there was no considerable difference in the mean change from DB-baseline [−25.6 (21.71) in DB-placebo group and −24.6 (20.40) in DB-blonanserin group], the mean change from OP-baseline was larger in DB-placebo group [−9.8 (16.39) in DB-placebo group and −4.0 (15.19) in DB-blonanserin group] (Table 3 and Fig. 3).

**Table 3.** Change from DB Baseline and OP Baseline in Positive and Negative Syndrome Scale Total Score

	Change from DB baseline						Change from OP baseline					
	DB-Placebo (*N* = 36)		DB-BNS (*N* = 70)		Overall (*N* = 106)		DB-Placebo (*N* = 36)		DB-BNS (*N* = 70)		Overall (*N* = 106)	
	*n*	Mean (SD)	*n*	Mean (SD)	*n*	Mean (SD)	*n*	Mean (SD)	*n*	Mean (SD)	*n*	Mean (SD)
OP baseline	36	−15.9 (17.43)	70	−20.6 (18.51)	106	−19.0 (18.21)						
Week 12	33	−24.6 (16.64)	62	−25.7 (19.98)	95	−25.3 (18.80)	33	−8.6 (12.08)	62	−4.1 (10.68)	95	−5.6 (11.34)
Week 28	28	−30.0 (19.75)	53	−25.7 (18.76)	81	−27.2 (19.10)	28	−11.0 (16.72)	53	−4.5 (10.98)	81	−6.7 (13.51)
Week 52	22	−32.5 (17.82)	41	−30.5 (18.07)	63	−31.2 (17.87)	22	−12.8 (16.00)	41	−8.1 (16.23)	63	−9.7 (16.18)
LOCF end point	36	−25.6 (21.71)	70	−24.6 (20.40)	106	−24.9 (20.76)	36	−9.8 (16.39)	69	−4.0 (15.19)	105	−6.0 (15.77)

Treatment group at placebo-controlled study: DB-Placebo = Placebo; DB-BNS = Blonanserin 8 or 16 mg; Overall = Placebo, Blonanserin 8 or 16 mg. DB baseline: Baseline of placebo-controlled study, OP baseline: day 1 of extension study.

LOCF, Last Observation Carried Forward; SD, standard deviation.

At the last assessment (LOCF), the mean change in PANSS subscale scores, PANSS 5-factor model scores, and CGI-S score was also maintained from OP-baseline without worsening similarly to that observed with the PANSS total score (Table 4).

**Table 4.** Change from DB Baseline and OP Baseline in Positive and Negative Syndrome Scale Subscale, Positive and Negative Syndrome Scale Five Factor Model, and Clinical Global Impressions-Severity of Illness Scale (Week 52, Last Observation Carried Forward)

	Change from DB baseline						Change from OP baseline					
	DB-Placebo (*N* = 36)		DB-BNS (*N* = 70)		Overall (*N* = 106)		DB-Placebo (*N* = 36)		DB-BNS (*N* = 70)		Overall (*N* = 106)	
	*n*	Mean (SD)	*n*	Mean (SD)	*n*	Mean (SD)	*n*	Mean (SD)	*n*	Mean (SD)	*n*	Mean (SD)
PANSS subscale												
Positive	36	−5.7 (6.01)	70	−6.3 (5.63)	106	−6.1 (5.74)	36	−2.2 (4.31)	69	−0.5 (4.13)	105	−1.1 (4.25)
Negative	36	−7.1 (5.67)	70	−6.1 (5.75)	106	−6.5 (5.72)	36	−3.4 (4.67)	69	−1.4 (4.17)	105	−2.1 (4.43)
General psychopathology	36	−12.8 (11.92)	70	−12.2 (10.54)	106	−12.4 (10.98)	36	−4.1 (9.51)	69	−2.1 (8.68)	105	−2.8 (8.98)
PANSS five factor												
Negative symptoms score	36	−6.5 (5.28)	70	−5.6 (5.48)	106	−5.9 (5.41)	36	−3.3 (4.44)	69	−1.0 (4.47)	105	−1.8 (4.57)
Excitement score	36	−2.8 (3.76)	70	−2.4 (3.30)	106	−2.5 (3.45)	36	−1.1 (2.88)	69	−0.3 (2.61)	105	−0.6 (2.72)
Cognitive disorders score	36	−3.7 (3.41)	70	−3.5 (3.20)	106	−3.6 (3.25)	36	−1.4 (2.74)	69	−0.6 (2.30)	105	−0.9 (2.48)
Positive symptoms score	36	−3.8 (3.98)	70	−4.3 (3.52)	106	−4.1 (3.67)	36	−1.4 (2.72)	69	−0.6 (2.56)	105	−0.8 (2.63)
Anxiety/depression score	36	−4.2 (4.93)	70	−4.0 (3.92)	106	−4.1 (4.27)	36	−1.3 (3.41)	69	−0.7 (3.16)	105	−0.9 (3.25)
CGI-S	36	−1.08 (1.296)	70	−1.17 (1.137)	106	−1.14 (1.188)	36	−0.39 (1.178)	69	−0.16 (0.908)	105	−0.24 (1.010)

Treatment group at placebo-controlled study: DB-Placebo = Placebo; DB-BNS = Blonanserin 8 or 16 mg; Overall = Placebo, Blonanserin 8 or 16 mg. DB baseline: Baseline of placebo-controlled study, OP baseline: day 1 of extension study.

CGI-S, Clinical Global Impressions-Severity of Illness Scale; PANSS, Positive and Negative Syndrome Scale; SD, standard deviation.

At OP-baseline, the percentage of PANSS responders showing ≥20%, ≥30%, ≥40%, and ≥50% improvement from DB-baseline was 69.8%, 50.9%, 36.8%, and 27.4%, respectively, whereas at the last assessment (LOCF), the responder rate increased to 75.5%, 70.8%, 57.5%, and 44.3%, respectively, after continued administration of blonanserin. PANSS score remission rate and CGI-I rate were also maintained or increased without worsening (Table 5).

**Table 5.** Analysis of Responder Rate in Positive and Negative Syndrome Scale Total Score, Remission Rate in Positive and Negative Syndrome Scale Total Score, and Improvement Rate in Clinical Global Impressions-Improvement Scale Score from DB Baseline

	DB-Placebo (*N* = 36)				DB-BNS (*N* = 70)				Overall (*N* = 106)			
	m	OP Baseline,* n *(%)	m	Week 52 (LOCF),* n *(%)	m	OP Baseline,* n *(%)	m	Week 52 (LOCF),* n *(%)	m	OP Baseline,* n *(%)	m	Week 52 (LOCF),* n *(%)
PANSS responder												
≥20% Improvement	36	23 (63.9)	36	28 (77.8)	70	51 (72.9)	70	52 (74.3)	106	74 (69.8)	106	80 (75.5)
≥30% Improvement	36	14 (38.9)	36	26 (72.2)	70	40 (57.1)	70	49 (70.0)	106	54 (50.9)	106	75 (70.8)
≥40% Improvement	36	10 (27.8)	36	20 (55.6)	70	29 (41.4)	70	41 (58.6)	106	39 (36.8)	106	61 (57.5)
≥50% Improvement	36	8 (22.2)	36	14 (38.9)	70	21 (30.0)	70	33 (47.1)	106	29 (27.4)	106	47 (44.3)
PANSS remission rate	36	20 (55.6)	36	26 (72.2)	70	49 (70.0)	69	52 (75.4)	106	69 (65.1)	105	78 (74.3)
CGI-I rate	36	7 (19.4)	36	18 (50.0)	69	25 (36.2)	68	27 (39.7)	105	32 (30.5)	104	45 (43.3)

*m* is the total number of subjects who did not discontinue before the corresponding visit and is used as the denominator to calculate percentages. PANSS responders are defined as patients with an improvement in PANSS total score from baseline value, where lower observed PANSS total scores indicate lower severity of schizophrenia. Responders are assessed at levels of improvement from baseline ≥20%, 30%, 40%, and 50%. PANSS remission rate is defined as the proportion of patients with grade 3 (mild) or less in all the following items: delusions (P1), conceptual disorganization (P2), hallucinatory behavior (P3), blunted affect (N1), passive/apathetic social withdrawal (N4), lack of spontaneity and flow of conversation (N6), mannerisms and posturing (G5), and unusual thought content (G9). CGI-I is defined as score of “very much improved” or “much improved” (CGI-I score of 1 or 2). Treatment group at placebo-controlled study: DB-Placebo = Placebo; DB-BNS = Blonanserin 8 or 16 mg; Overall = Placebo, Blonanserin 8 or 16 mg. DB baseline: Baseline of placebo-controlled study, OP baseline: day 1 of extension study.

CGI-I, Clinical Global Impressions-Improvement Scale; LOCF, Last Observation Carried Forward; PANSS, Positive and Negative Syndrome Scale.

### Pharmacokinetics

The plasma concentrations 2–4 hours after blonanserin administration (approximate peak) at weeks 28 and 52 were about twice the approximate trough concentrations, and the approximate trough plasma concentrations at weeks 28 and 52 both increased in a dose-dependent manner. The plasma concentration of the metabolite M-1 was approximately the same as that of blonanserin and also increased in a dose-dependent manner.

### Safety

The overall incidence of adverse events was 90.6% (96 subjects), and the incidence did not greatly differ between the three groups allocated in the placebo-controlled study (Table 6). The adverse events with a high incidence ≥10% comprised nasopharyngitis (35.8%), worsening schizophrenia (19.8%), akathisia (17.9%), headache (17.0%), tremor (17.0%), vomiting (14.2%), weight increased (14.2%), acne (14.2%), dystonia (11.3%), constipation (10.4%), and skin abrasion (10.4%) (Table 7).

**Table 6.** Summary of Adverse Events

	DB-Placebo (*N* = 36)	DB-BNS (*N* = 70)	Overall (*N* = 106)
AEs	33 (91.7)	63 (90.0)	96 (90.6)
Mild	18 (50.0)	43 (61.4)	61 (57.5)
Moderate	12 (33.3)	14 (20.0)	26 (24.5)
Severe^a^	3 (8.3)	6 (8.6)	9 (8.5)
Schizophrenia	3 (8.3)	3 (4.3)	6 (5.7)
Completed suicide	0	2 (2.9)	2 (1.9)
Aggression	0	1 (1.4)	1 (0.9)
Suicide attempt	0	1 (1.4)	1 (0.9)
Death	0	2 (2.9)	2 (1.9)
Completed suicide	0	2 (2.9)	2 (1.9)
Serious	8 (22.2)	9 (12.9)	17 (16.0)
Pharyngitis streptococcal	0	1 (1.4)	1 (0.9)
Toxicity to various agents	1 (2.8)	0	1 (0.9)
Schizophrenia	7 (19.4)	3 (4.3)	10 (9.4)
Completed suicide	0	2 (2.9)	2 (1.9)
Impulsive behavior	0	1 (1.4)	1 (0.9)
Suicidal ideation	0	1 (1.4)	1 (0.9)
Suicide attempt	0	1 (1.4)	1 (0.9)
AE leading to discontinuation of study drug	4 (11.1)	5 (7.1)	9 (8.5)
AE leading to dose decrease of study drug	12 (33.3)	12 (17.1)	24 (22.6)
AE leading to dose increase of study drug	1 (2.8)	9 (12.9)	10 (9.4)
AE related to Extrapyramidal syndrome^b^	17 (47.2)	24 (34.3)	41 (38.7)
AE related to Prolactin increased^c^	9 (25.0)	15 (21.4)	24 (22.6)
Weight increased	4 (11.1)	11 (15.7)	15 (14.2)
Weight decreased	1 (2.8)	0	1 (0.9)

All AEs were coded using MedDRA dictionary version 21.1.

^a^ A patient may have had two or more AEs, thus the same patient may appear in different AEs in the following breakdown.

^b^ Patients with any Extrapyramidal syndrome AE such as oculogyric crisis, salivary hypersecretion, muscle rigidity, akathisia, tremor, dystonia, dyskinesia, bradykinesia, extrapyramidal disorder, myoclonus, or parkinsonian gait.

^c^ Patients with any Prolactin increased AE such as hyperprolactinemia, blood prolactin increased, or galactorrhea.

AE, adverse event.

**Table 7.** Common Adverse Events (≥5% Incidence)

System organ class	DB-Placebo	DB-BNS	Overall
Preferred term,* n *(%)	(*N* = 36)	(*N* = 70)	(*N* = 106)
Endocrine disorders			
Hyperprolactinemia	4 (11.1)	6 (8.6)	10 (9.4)
Eye disorders			
Conjunctivitis allergic	4 (11.1)	4 (5.7)	8 (7.5)
Gastrointestinal disorders			
Vomiting	5 (13.9)	10 (14.3)	15 (14.2)
Constipation	4 (11.1)	7 (10.0)	11 (10.4)
Diarrhea	3 (8.3)	7 (10.0)	10 (9.4)
Abdominal pain	5 (13.9)	4 (5.7)	9 (8.5)
Nausea	2 (5.6)	7 (10.0)	9 (8.5)
Dental caries	2 (5.6)	5 (7.1)	7 (6.6)
Abdominal discomfort	3 (8.3)	3 (4.3)	6 (5.7)
Infections and infestations			
Nasopharyngitis	17 (47.2)	21 (30.0)	38 (35.8)
Injury, poisoning, and procedural complications			
Skin abrasion	5 (13.9)	6 (8.6)	11 (10.4)
Wound	3 (8.3)	5 (7.1)	8 (7.5)
Arthropod sting	2 (5.6)	5 (7.1)	7 (6.6)
Contusion	1 (2.8)	5 (7.1)	6 (5.7)
Investigations			
Weight increased	4 (11.1)	11 (15.7)	15 (14.2)
Blood prolactin increased	4 (11.1)	6 (8.6)	10 (9.4)
Blood creatine phosphokinase increased	2 (5.6)	4 (5.7)	6 (5.7)
Musculoskeletal and connective tissue disorders			
Back pain	2 (5.6)	6 (8.6)	8 (7.5)
Myalgia	3 (8.3)	5 (7.1)	8 (7.5)
Pain in extremity	2 (5.6)	4 (5.7)	6 (5.7)
Nervous system disorders			
Akathisia	6 (16.7)	13 (18.6)	19 (17.9)
Headache	6 (16.7)	12 (17.1)	18 (17.0)
Tremor	9 (25.0)	9 (12.9)	18 (17.0)
Dystonia	5 (13.9)	7 (10.0)	12 (11.3)
Somnolence	4 (11.1)	6 (8.6)	10 (9.4)
Psychiatric disorders			
Schizophrenia	10 (27.8)	11 (15.7)	21 (19.8)
Respiratory, thoracic, and mediastinal disorders			
Oropharyngeal pain	2 (5.6)	7 (10.0)	9 (8.5)
Epistaxis	1 (2.8)	5 (7.1)	6 (5.7)
Skin and subcutaneous tissue disorders			
Acne	8 (22.2)	7 (10.0)	15 (14.2)
Eczema	7 (19.4)	3 (4.3)	10 (9.4)
Pruritus	2 (5.6)	5 (7.1)	7 (6.6)

All adverse events were coded using MedDRA dictionary version 21.1.

Most of the adverse events were mild or moderate in severity, whereas severe adverse events occurred in nine patients (8.5%). During the study, two patients died because of the adverse events; both events were completed suicide, but a causal relationship with the study drug was ruled out. Serious adverse events, including deaths, occurred in 17 patients (16.0%). Of these, the causal relationship with the study drug could not be ruled out in one patient who developed worsening schizophrenia. Adverse events led to discontinuation of the study drug in nine patients (8.5%) (Table 6).

The incidence of extrapyramidal adverse events was 38.7%. Of these, the incidence of events that warranted reduction in blonanserin dose was 11.3%, but there were no serious events, no events leading to discontinuation, and no events judged to be severe. The most common extrapyramidal adverse events were akathisia (17.9%), tremor (17.0%), dystonia (11.3%), and dyskinesia (4.7%), and most of these events occurred within the first 6 months of this study. Tardive dyskinesia did not occur. The change in total DIEPSS score at the last assessment (LOCF) [mean (SD)] was 0.08 (1.261), which would not present a noteworthy clinical problem. The incidence of prolactin-related adverse events was 22.6%, including hyperprolactinemia (9.4%), increased blood prolactin (9.4%), irregular menstruation (1.9%), amenorrhea-galactorrhea syndrome (0.9%), and galactorrhea (0.9%) (Table 6); however, there were no events leading to dose reduction or discontinuation of the study drug.

There was almost no change in CGI-SS score at the last assessment (LOCF), which was 0.05 (SD, 0.352). Only four patients (3.8%) presented with score deterioration by the last assessment. There was no considerable increase in suicide risk.

No clinically relevant changes were observed in body weight and height, z-scores and percentiles of weight and height, or BMI after treatment compared with DB-baseline (Table 8). The mean change in weight over time from DB-baseline is shown in Table 9. Weight-related adverse events comprised weight increased in 15 patients (14.2%) and weight decreased in 1 patient (0.9%) (Table 6). During the study drug treatment period, 14 patients experienced weight increased of at least 7% compared with DB-baseline. There were no clinically relevant changes in laboratory test values, including metabolic parameters, vital signs, electrocardiographic parameters, or prolactin levels (Table 8).

**Table 8.** Change from DB Baseline in Metabolic and Laboratory Parameters (Week 52, Last Observation Carried Forward)

	DB-Placebo (*N* = 36)		DB-BNS (*N* = 70)		Overall (*N* = 106)	
	*n*	Mean (SD)	*n*	Mean (SD)	*n*	Mean (SD)
Weight (kg)	36	2.65 (5.510)	70	2.81 (5.350)	106	2.75 (5.379)
z-Score of weight	36	0.3010 (0.63478)	70	0.2835 (0.60333)	106	0.2895 (0.61122)
Percentile of weight	36	6.81 (19.845)	70	4.76 (14.866)	106	5.46 (16.657)
Height (cm)	36	0.89 (1.324)	70	0.75 (2.097)	106	0.80 (1.865)
z-Score of height	36	0.1332 (0.20444)	70	0.0817 (0.33119)	106	0.0992 (0.29430)
Percentile of height	36	4.12 (6.054)	70	3.14 (8.519)	106	3.48 (7.754)
BMI (kg/m^2^)	36	0.82 (2.044)	70	0.87 (1.844)	106	0.85 (1.905)
Glucose (mg/dL)^a^	36	2.6 (9.76)	67	2.2 (10.27)	103	2.3 (10.05)
Hemoglobin A1c (%)^a^	36	−0.04 (0.206)	68	−0.02 (0.239)	104	−0.03 (0.228)
Triglycerides (mg/dL)^a^	36	10.1 (76.27)	67	1.3 (58.63)	103	4.4 (65.10)
Total cholesterol (mg/dL)^a^	36	7.2 (25.69)	67	−3.0 (27.02)	103	0.5 (26.88)
Prolactin (μg/L)^a^						
Females	22	−10.642 (37.7317)	37	−1.565 (36.5602)	59	−4.949 (36.9421)
Males	14	8.134 (20.5599)	30	−10.243 (31.2829)	44	−4.396 (29.3728)

Treatment group at placebo-controlled study: DB-Placebo = Placebo; DB-BNS = Blonanserin 8 or 16 mg; Overall = Placebo, Blonanserin 8 or 16 mg. DB baseline: Baseline of placebo-controlled study.

^a^ Fasting conditions.

BMI, body mass index; SD, standard deviation.

**Table 9.** Change from DB Baseline in Weight (kg)

	DB-Placebo (*N* = 36)		DB-BNS (*N* = 70)		Overall (*N* = 106)	
	*n*	Mean (SD)	*n*	Mean (SD)	*n*	Mean (SD)
OP baseline	36	0.18 (2.437)	70	0.49 (2.984)	106	0.39 (2.802)
Week 4	36	0.29 (2.662)	66	0.88 (3.570)	102	0.67 (3.277)
Week 8	34	1.08 (3.216)	66	1.41 (3.891)	100	1.30 (3.662)
Week 12	33	1.44 (3.761)	62	1.52 (3.826)	95	1.49 (3.784)
Week 28	28	2.96 (4.593)	53	2.60 (4.379)	81	2.72 (4.429)
Week 52	22	3.04 (5.595)	41	3.65 (5.723)	63	3.43 (5.641)
LOCF end point	36	2.65 (5.510)	70	2.81 (5.350)	106	2.75 (5.379)

Treatment group at placebo-controlled study: DB-Placebo = Placebo; DB-BNS = Blonanserin 8 or 16 mg; Overall = Placebo, Blonanserin 8 or 16 mg. DB baseline: Baseline of placebo-controlled study.

LOCF, Last Observation Carried Forward; SD, standard deviation.

## Discussion

We certified the efficacy, safety, and tolerability of long-term blonanserin administration, 4–24 mg/day for 52 weeks, in patients with adolescent schizophrenia who completed the preceding placebo-controlled study and continued in the present extension study.

Demographics and characteristics of patients who transferred to the extension study did not greatly differ from those in the preceding placebo-controlled study. The continuation rate of blonanserin treatment in the present extension study was 75.5% at week 24 (168 days), and 63 patients (57.8%) completed the study. The remission rate at the last assessment (LOCF) compared with OP-baseline in this study increased in the DB-placebo group (55.6%–72.2%) and was maintained in pooled DB-blonanserin tablet group (70.0%–75.4%). The continuation rates reported for atypical antipsychotics administered to patients with adolescent schizophrenia in open-label long-term treatment study were ∼75%, with 6-month administration of risperidone, paliperidone, and aripiprazole (Pandina et al. 2012; Savitz et al. 2015) and ∼50% with 1-year administration of risperidone (Pandina et al. 2012). In a study of long-term blonanserin administration in patients with adult schizophrenia (percentage receiving monotherapy, 72.1%), the percentage of subjects who completed the study was 78.7% at week 28 (182 days) and 62.3% at or after week 52 (364 days) (Murasaki 2007b). *Post hoc* analysis of the long-term study revealed that if discontinuation rate and remission rate were used as indicators, blonanserin treatment would prove itself effective in patients with adult schizophrenia (Ishigooka and Nakamura 2011). In adolescents with schizophrenia, the continuation rate of blonanserin treatment was not lower compared with other atypical antipsychotics, and a similar level of effectiveness might be expected with blonanserin treatment in adolescents to that in adults.

From the results of the placebo-controlled study, the efficacy of 6 weeks of blonanserin administration at 8 or 16 mg/day was similar to that of other atypical antipsychotics approved for indication of adolescent schizophrenia (clinical study No.: Japic CTI-111724). In DB-blonanserin group, the mean change in PANSS total score at the last assessment (LOCF) in the present extension study was −24.6 from DB-baseline and −4.0 from OP-baseline. The effect of blonanserin confirmed in the placebo-controlled study (−20.6) was sustained with long-term administration. However, in DB-placebo group, which started blonanserin treatment at 4 mg/day in a nearly drug-naive state after receiving no antipsychotics, including blonanserin during the treatment period (6 weeks) and the transition period (maximum 2 weeks) of the placebo-controlled study, PANSS total score decreased to −25.6 from DB-baseline, and the change was similar to that in DB-blonanserin group after 1 year of administration. The larger change observed in DB-placebo group from OP-baseline (−9.8) than that in DB-blonanserin group (−4.0) was presumably because of the presence or absence of the effect of actual drug during the placebo-controlled study. In both DB-placebo and DB-blonanserin groups, the mean change at the last assessment (LOCF) in PANSS subscale/5-factor model scores and in CGI-S score showed a maintenance/improvement effect similar to that seen in PANSS total score. Because almost half (44.3%) of the patients showed ≥50% improvement in PANSS, which has been reported to correspond with moderate improvement or better (Leucht et al. 2017), it is considered that the efficacy is similar to that of other atypical antipsychotics (Savitz et al. 2015).

The discontinuation rate due to adverse events was 3.7%, which did not greatly differ from the rates reported for other antipsychotics (5.1%–9.2%) (Pandina et al. 2012; Findling et al. 2015). Considering the continuation rate in 1-year treatment of blonanserin (57.8%), there were no major tolerability issues with long-term clinical use of blonanserin. In general, the risk of extrapyramidal symptoms, metabolic adverse reactions, weight increased, and other adverse effects with antipsychotics is higher in adolescent patients than in adults (Correll et al. 2011). Nevertheless, with blonanserin administration, the incidence of extrapyramidal adverse events (37.8%), which are thought to occur with higher frequency and dose dependently, was similar to or lower than that in Japanese 52-week open-label studies for blonanserin oral tablet in adults (35.8%; 52.5%) (Murasaki 2007b; Kinoshita 2008); moreover, such events did not become serious, lead to study withdrawal, or include tardive dyskinesia in this study. In addition, according to a network meta-analysis of Japanese randomized-controlled studies on antipsychotics for adult schizophrenia, the risk of blonanserin for extrapyramidal adverse events was almost comparable with other second-generation antipsychotics (higher than olanzapine and quetiapine but similar to aripiprazole, clozapine, paliperidone, perospirone, and risperidone), whereas the risk was significantly lower than haloperidol (Kishi et al. 2017). Based on these results, it was speculated that the incidence of extrapyramidal adverse events with long-term blonanserin treatment in adolescent patients was similar to that with other second-generation antipsychotics. Prolactin did not increase considerably from DB-baseline, and all cases of adverse events related to prolactin increase were deemed to be mild. Therefore, it was concluded that adolescent patients show a similar safety profile of blonanserin to that confirmed in adult patients, that is, the risk of extrapyramidal adverse events is lower than that with haloperidol (Murasaki 2007a; Harvey et al. 2019) and the risk of prolactin increase is lower than that with risperidone (Miura 2008). Metabolic parameters and weight increased are important factors affecting the selection of atypical antipsychotics. The administration of blonanserin led to almost no changes in metabolic parameters from DB-baseline. In addition, the mean change from DB-baseline in weight and height at the last assessment (LOCF) was 2.75 kg and 0.80 cm, respectively. Accounting for developmental growth based on the Survey of School Health Statistics (Portal Site of Official Statistics in Japan 2019), the mean change in weight and height z-scores (weight, 0.2895; height, 0.0992) or percentiles (weight, 5.46; height, 3.48) observed with blonanserin treatment might reflect minimal adverse effects on weight and height of adolescent patients with schizophrenia. Furthermore, given that weight gain was gradual and weight-related adverse events were mild, it is reasonable to conclude that such events would not have a notable effect on weight and height of adolescents.

In this extension study continued from the preceding placebo-controlled study, the administration started at 4 mg/day, and the modal and maximum daily doses of oral blonanserin were mostly within the range of 8–16 mg, which correspond to the maintenance dose in adults. The daily dose was less than 8 mg in more than 20% of patients, and few patients needed more than 16 mg. These results support the evidence of the preceding study, in which blonanserin was started at 4 mg/day and the dose was titrated until achieving the optimal range (8–16 mg/day, corresponding to maintenance dose in adults) in which superior efficacy of blonanserin to placebo was demonstrated. In adults, starting the treatment with the approved daily dosage of 8 mg blonanserin is important to achieve an early response to treatment (Tsuchimori et al. 2019). However, oral blonanserin administration was started at 4 mg/day both in this study and in the placebo-controlled study, and the incidence of adverse events increased with increasing dose of oral blonanserin. Considering less evidence in the treatment with oral blonanserin in adolescent schizophrenia, the initial daily dosage of 4 mg was considered to be appropriate.

We confirmed that when oral blonanserin was used at a range of 4–16 mg/day in patients with adolescent schizophrenia, it was possible to achieve sufficient effectiveness and there was less considerable effect on weight increased or metabolic parameters, which require attention especially in adolescents. In addition, no major safety issues were seen in patients receiving blonanserin at more than 16 mg/day in this study. Long-term treatment with high doses of multiple antipsychotics can trigger dopamine supersensitivity psychosis, which can make cases intractable; however, long-term blonanserin use in patients with dopamine supersensitivity psychosis could reportedly allow a greater reduction of antipsychotic dosage than olanzapine (Niitsu et al. 2020). In long-term drug treatment of schizophrenia, oral blonanserin at 4–16 mg/day may be a promising treatment option to allow seamless therapy from adolescence to adulthood.

### Limitations

This study was a long-term study of blonanserin administration continued from the preceding placebo-controlled study; hence, the patients who withdrew from the placebo-controlled study and patients who did not choose to transfer to the present extension study were not included. To generalize the evidence for long-term blonanserin treatment for adolescent schizophrenia, further study is required with various patient backgrounds or under the conditions of actual clinical use. Due to the open-label design of the study, all the patients received blonanserin with no comparator, and the potential bias should be assumed for the interpretation of the results.

## Conclusions

This long-term extension study showed that 52 weeks of oral blonanserin treatment improved or stabilized psychiatric symptoms in patients with adolescent schizophrenia. There were no major issues with the safety or tolerability of blonanserin administration in this study. Considering relatively less adverse effects on weight increased and metabolic parameters, blonanserin is expected to be a safe/tolerable treatment option for adolescent schizophrenia that can be used seamlessly from adolescence to adulthood.

## Clinical Significance

Fifty-two weeks of oral blonanserin treatment improved or stabilized psychiatric symptoms in patients with adolescent schizophrenia. There were no major issues with the safety or tolerability of blonanserin administration in this long-term extension study. Thus, blonanserin is expected to be a safe/tolerable treatment option for adolescent schizophrenia, which can be used seamlessly from adolescence to adulthood.

## Disclaimer

Sumitomo Dainippon Pharma makes individual patient, deidentified data sets and associated clinical documents such as study protocol, statistical analysis plan, and clinical study report available upon request through the Clinical Study Data Request site (https://www.clinicalstudydatarequest.com/Study-Sponsors.aspx) within 12 months of posting the study results on clinicaltrials.gov Access is provided after a research proposal is submitted and has received approval from the Independent Review Panel and after a Data Sharing Agreement is in place. Access is provided for an initial period of 12 months but an extension can be granted, when justified, for up to another 12 months.
